# Five-year monitoring of a gay-friendly voluntary counselling and testing facility in Switzerland: who got tested and why?

**DOI:** 10.1186/1471-2458-12-422

**Published:** 2012-06-08

**Authors:** Cédric Gumy, André Jeannin, Hugues Balthasar, Thérèse Huissoud, Vincent Jobin, Michael Häusermann, Hubert Crevoisier, Philippe Sudre, Françoise Dubois-Arber

**Affiliations:** 1 Institute of Social and Preventive Medicine (IUMSP), Lausanne University Hospital, Lausanne, Switzerland; 2 Swiss Aids Federation, Zurich, Switzerland; 3 Dialogai, Geneva, Switzerland; 4 General Directorate of Health, Department of Regional Affairs, Economy and Health (DARES), Geneva canton, Switzerland; 5 Institute of Social and Preventive Medicine, Bâtiment Biopôle 2, Rte de la Corniche 10, CH-1010, Lausanne, Switzerland

**Keywords:** Anonymous HIV counselling and testing, Homosexuality, Gay-friendly facility, Monitoring, Switzerland

## Abstract

**Background:**

An increase in new HIV cases among men who have sex with men (MSM) has been reported in Switzerland since 2001. A rapid result HIV testing for MSM through voluntary counselling and testing (VCT) facility (“Checkpoint”) was opened in Geneva in 2005. This gay-friendly facility, the first to open in Switzerland, provides testing for sexually transmitted infections (STI) and rapid result HIV testing and counselling. Our objective was to analyze Checkpoint’s activity over its first five years of activity and its ability to attract at-risk MSM.

**Methods:**

We used routine data collected anonymously about the facility activity (number of clients, number of tests, and test results) and about the characteristics of the clientele (sociodemographic data, sexual risk behaviour, and reasons for testing) from 2005 to 2009.

**Results:**

The yearly number of HIV tests performed increased from 249 in 2005 to 561 in 2009. The annual proportion of positive tests among tests performed varied between 2% and 3%. Among MSM clients, the median annual number of anal intercourse (AI) partners was three. Roughly 30% of all MSM clients had at least one unprotected anal intercourse (UAI) experience in the previous 12 months with a partner of different/unknown HIV status.

The main reason for testing in 2007, 2008, and 2009 was “sexual risk exposure” (~40%), followed by “routine” testing (~30%) and “condom stopping in the beginning of a new steady relationship” (~10%). Clients who came to the facility after a sexual risk exposure, compared to clients who came for "routine testing" or "condom stopping" reasons, had the highest number of AI partners in the previous 12 months, were more likely to have had UAI with a partner of different/unknown HIV status in the previous 12 months (respectively 57.3%, 12.5%, 23.5%), more likely to have had an STI diagnosed in the past (41.6%, 32.2%, 22.9%), and more likely to report recent feelings of sadness or depression (42.6%; 32.8%, 18.5%).

**Conclusion:**

Many of Checkpoint's clients reported elevated sexual risk exposure and risk factors, and the annual proportion of new HIV cases in the facility is stable. This VCT facility attracts the intended population and appears to be a useful tool contributing to the fight against the HIV epidemic among MSM in Switzerland.

## Background

As in most Western European countries, Switzerland has observed a progression of the HIV epidemic in men who have sex with men (MSM) in recent years [1]. Between 2003 and 2008, the annual number of new HIV cases reported among MSM doubled, from 160 to 327 [2], but it decreased or remained stable among the other transmission groups. In 2009, 43.9% of new HIV-positive cases were related to MSM (heterosexuals: 45.4%, injecting drug users (IDU): 4.8%) [3]. In a survey conducted in 2009 among a convenience sample (offline and online) of MSM living in Switzerland, the reported prevalence of HIV among MSM in Switzerland was 6.4% among online respondents and 10.2% among offline respondents [4]. Among the Swiss respondents of the European MSM Internet Survey (EMIS) in 2010, the reported prevalence was 9.0% [5].

Sexual risk behaviours, in particular unprotected anal intercourse (UAI), i.e. without a condom, have increased lately in Switzerland [6] and in many European countries [6-12]. In addition, people living with HIV live longer and in better health than previously, with a potential increase in the risk of sexual HIV transmission and increased HIV prevalence.

For these reasons, as well as the persistence of high levels of risk behaviour in this group, MSM in particular are exposed to sexual HIV transmission in Switzerland.

Moreover, data on MSM attending voluntary counselling and testing (VCT) centres suggest that high rates of HIV infection may be caused by HIV transmission during the acute phase of infection by persons who are highly contagious [13] and do not know it [14]. In Switzerland, the proportion of recent infections among newly HIV-positive diagnosed MSM was 41.3% in 2009 [3].

Among participants in a repeated cross-sectional survey conducted in convenience samples (using gay websites, gay newspapers and gay organizations recruitment) of MSM living in Switzerland, it was found that a high proportion (90%) of respondents have ever been tested, roughly 40% in the previous 12 months in 2009 [4]. However, the quality of counselling seems to be unevenly distributed. More than half of MSM who underwent testing were tested at their physician’s office and, often, did not receive comprehensive MSM-specific counselling. At the physician’s office, only 31.5% received pre-test counselling and 22.2% post-test counselling [4].

MSM-specific counselling is needed as some MSM may feel reluctant to undergo testing for HIV in common testing centres and talk about their sexual identity and practices. Moreover, some physicians and nurses may feel unprepared for dealing with MSM clients. Having a gay-friendly physician is an important desire expressed by MSM, but it is rarely fulfilled. Two unpublished studies reported by Häusermann et al. [15] conducted among convenience samples of MSM in Switzerland found that approximately half of the participants who were tested for HIV in 2002 did not have any MSM-specific counselling before or after testing.

In December 2003, the Federal Office of Public Health (FOPH) elaborated 12 objectives in order to fight the spread of HIV. One of the objectives addressed personal prevention strategies through VCT implementation [16]. In this context, in January 2005, a local gay NGO in Geneva opened the VCT facility called “Checkpoint” [17] to offer rapid result anonymous HIV and sexually transmitted infection (STI) testing to MSM.

Checkpoint was inspired by the same-named facility opened in Amsterdam [18] two years earlier. Since that time, other similar facilities have opened in Europe: in Munich and Barcelona [19] in 2007, Paris in 2010, and Zurich in 2006 [20]

### Checkpoint operation

Checkpoint is an “open door” facility primarily addressing gays and MSM, offering anonymous rapid result HIV tests (results available within 20 minutes) and combined tests (antigen, antibody). Currently, regarding STI, a "package" including rapid test for HIV, syphilis and Hepatitis C is systematically proposed. The status regarding Hepatitis B immunization is systematically checked and immunization proposed, if needed. For the other STI, testing is proposed according to sexual history and reported symptoms or on demand. The facility is run by a gay nurse, with the help of volunteers, under the medical supervision of the Geneva University Hospital (GUH). Initially reserved to MSM only, the organization of the consultation and policy concerning non-MSM changed in 2007. Since that time, non-MSM clients have been accepted and it is possible to visit without appointment. Checkpoint is open 8 hours a week. In 2005 and 2006, it was opened on Monday evening (4 h) and Tuesday afternoon (4 h); from 2007 onwards, both days, 4 hours in the evening. An in-house monitoring system of the services delivered by the facility and of the client’s characteristics was set up after its opening.

The consultation is confidential and anonymous; people may provide any name or pseudonym for record at entry. An identification number is attributed to each client in order to match the consultation with the test result. Each test request is considered independently from possible previous request. Even if a previous test was performed, the complete procedure is followed and the client is considered as a new client because the situation of the client (risk exposure episodes, relationship or single status) may have changed and a new analysis of the client’s situation must be conducted. At arrival, clients fill out a computerized entry questionnaire about personal test history, socio-demographic data, sexual behaviours, and sexual risk taking, which is used as the basis of the pre-test counselling by the nurse who performs the test (Abbott’s Determine® HIV-1/2 AG/AB as of September 2009). If the result of the test is reactive, a confirmatory test (Immunoblot or Western-blot) is done at the virology laboratory of the GUH. If the test is not reactive, a simple post-test counselling is offered. If the blood sample is confirmed positive for HIV (result available after one week), diagnosis is given by the staff’s doctor, extensive post-test counselling as well as psychological support is provided, and the client is referred to a medical care service. On the day of the consultation, if the test is reactive, as well as after a confirmed positive test, psychological support is offered to the client by a mentor who is an HIV-positive MSM worker of a neighbouring HIV-prevention structure. The client is also proposed to join a programme of support (workshop) intended for newly infected MSM in Switzerland (“Queer+”).

In pre- and post-test counselling, prevention messages are individualized according to the needs of each client and special attention is given to mental health and psychoactive substance use (alcohol, illegal drugs) before and during sex. STI testing (generally not anonymous) is done for Hepatitis A (HAV), B (HBV) and C (HCV), syphilis, HPV, and anal, genital and pharyngeal gonorrhoea and chlamydia. HAV and HBV vaccinations are also proposed separately or in parallel to HIV testing.

Although MSM-specific VCT facilities have existed for several years in the United States [21], they are recent developments in European, African [22] and Asian countries [23], and little is known about the characteristics (levels of sexual risk exposure, reason for the test) of the clientele frequenting these VCT facilities.

The first objective of this observational study that used routinely collected information as part of the counselling activity was to evaluate Checkpoint’s activity over its first five years of operation. The second objective was to assess whether this type of facility is able to attract the most at-risk portion of the MSM population by analyzing the characteristics of its clientele, particularly the risk profiles and reasons for testing. This analysis was performed in the context of the continuous evaluation of the facility.

## Methods

The present data were collated for monitoring purposes in the context of an evaluation contract from the General Directorate of Health, Department of Regional Affairs, Economy, and Health (DARES) of Geneva canton. We conducted a descriptive secondary analysis of data routinely collected from all clients at the facility from January 2005 to December 2009. We used data on the activity of the facility, such as the annual number of consultations, number of HIV tests performed, and number of HIV-positive tests, and data from the computerized questionnaire filled out anonymously by each client. Most of the questions used in this short questionnaire come from the MSM survey included in the HIV behavioural surveillance system in Switzerland [24]. At the facility, an identification number is associated with each client at their first visit. If the same client returns after a delay of 6 months or more, a new identification number is assigned because he is considered a new client. Therefore, one client may visit the VCT facility twice a year with two identification numbers. The number of people who were in this situation during the study period is not known.

We used the following information from the entry questionnaire:

1. Socio-demographics : age, sex, sexual orientation (gay/homosexual, bisexual, heterosexual), current relationship status with a male partner (“Are you currently in a steady relationship with a male partner?”) and HIV serostatus of the current steady partner (only for those who have a steady male partner);

2. Sexual activity: number of anal intercourse (AI) partners (gender not specified) in the previous 12 months;

3. Risk: three measures of sexual risk exposure were considered: UAI with at least one steady partner in the previous 12 months, UAI with at least one casual partner in the previous 12 months, and the occurrence (yes/no) of UAI with a partner (gender not specified) of different/unknown HIV status in the previous 12 months (“in the last 12 months, have you ever practiced at least once UAI with a partner of different or unknown HIV status?” [25]);

4. Testing history: new or returning client, number of previous HIV tests, and reasons for current visit; for an HIV test, the following list of reasons are offered, one or more of which may be selected: sexual risk exposure (receptive/insertive anal penetration, sperm/blood in mouth, condom failure, other type of risk exposure); "routine" testing; desire to stop using a condom in the beginning of a new steady relationship; STI test; other;

5. Depressive symptoms and psychoactive drugs use: feelings of sadness/depression in the previous 4 weeks (this item is used to introduce the topic of mental health in the interview) and frequency of alcohol and drug consumption (before and during sexual intercourse).

The question about the reason for testing for HIV has been asked since 2007. Because each client could cite more than one reason, we used an algorithm to determine which one to keep as the main reason for testing in the case of multiple responses. Among MSM (n = 1072), 86% gave one reason, 11% gave two reasons, 0.6% gave three reasons, and 2.3% gave no reason. Priority was attributed to “sexual risk exposure”, followed by “condom stopping in the beginning of a new steady relationship”, then “routine”, “STI”, and finally “other reason”. We concentrated on comparing the first three groups (“sexual risk exposure”, “routine testing” and “condom stopping in the beginning of a new steady relationship”) because they are the most relevant and include the vast majority of clients.

We calculated the ratio of the number of positive tests detected at Checkpoint among MSM between 2005 and 2009 over the total number of new positive tests detected among self-declared MSM living in the canton of Geneva in the same time frame.

### Analysis

Based on the visit as unit of analysis, according to Checkpoint procedures, we first analyzed the results of the overall testing activity of the facility and socio-demographic profile of its clientele as a whole. Then, we restricted the subsequent analysis to the population of self-identified MSM (i.e. men who declared sexual intercourse with men or with men and women, independently of sexual identity or orientation).

We constructed five groups according to the main reason for testing: “sexual risk exposure”, “routine”, “condom stopping in the beginning of a new steady relationship”, “STI”, and “other”. We compared these groups in terms of “lifestyle” and sexual practices; to increase statistical power, data available in 2007, 2008, and 2009 was pooled. Chi-square tests were used for categorical variables and K-sample median tests for numerical variables. We set the overall significance threshold at *p* < 0.05, by reducing it to *p* < 0.00555 for each individual test after Bonferroni correction. Missing values were not considered because we used complete case analysis on a pairwise basis, except for the question of UAI with a partner of different/unknown HIV status, where the denominator is all MSM clients. Percentages of missing values were <5% except when indicated in the tables. We analyzed the data with PASW statistic software version 18 for Windows.

## Results

### Facility activity

The annual number of clients visiting the facility more than doubled between 2005 and 2009, as did the number of HIV tests performed (Table 1). The annual number of positive HIV tests increased from 6 to 11. The prevalence of newly diagnosed positive HIV tests among all tests performed (all but one were MSM among newly infected people) remained stable at approximately 2.0%, except in 2007 (3.0%).

**Table 1:** Testing activity at Checkpoint, 2005-2009

	**2005**	**2006**	**2007**	**2008**	**2009**
Number of clients	245	251	313	475	574
Number of HIV tests	249	282	328	480	561
Number of positive tests	6	6	10	10	11
Proportion of positive HIV tests among tests (%)	2.4	2.1	3.0	2.1	1.9

Note: All but one positive test (in 2008) concerned MSM and were newly detected positive tests, i.e. tests regarding clients who had never been tested positive before.

The median age of the clients each year was between 31 and 34 years of age (Table 2). Most clients were male (between 86.4% and 99.4%). Among male clients, 64.1-78.1% identified themselves as homosexuals, 7.5-17.0% as bisexuals, and 4.9-22.0% as heterosexuals. The proportion of self-identified MSM varied between 78.4% and 96.4% of all male clients and decreased over the years presumably due to changes in the policy and opening hours of the facility.

**Table 2:** Socio-demographic profile of Checkpoint clientele, 2005-2009

	**2005**	**2006**	**2007**	**2008**	**2009**
	**(n = 245)**	**(n = 251)**	**(n = 313)**	**(n = 475)**	**(n = 574)**
**Median age,years (SD)**	32 (9.9)	34 (11.2)	31 (11.6)	32 (10.7)	31.5 (10.6)
**Sex**					
Male (%)	94.7	98.4	99.4	90.1	86.4
Female (%)	5.3	1.6	0.6	9.9	13.6
**Sexual orientation of males (%)**					
Gay/homosexual	77.2	78.1	77.8	82.2	64.1
Bisexual	11.6	17.0	16.4	7.5	13.9
Heterosexual	11.2	4.9	5.8	10.3	22.0
**Men reporting having sex with men (%)**	90.9	96.4	95.2	90.4	78.4

The proportion of MSM in a steady relationship with another man at the time of the test was stable over time. In 2009, 51.9% of MSM were in such a relationship (Table 3). Among these men, roughly 11% had an HIV-positive partner. This proportion was stable over the years except in 2007 (5.6%). The serostatus of the partner was unknown or the information was missing for 26.4% of the MSM in relationships.

**Table 3:** **Characteristics of MSM**^**1**^**Checkpoint clients, 2005-2009**

	**2005**	**2006**	**2007**	**2008**	**2009**
**Number of clients**	211	238	296	387	389
**Median age (years)**	33	34	31	33	32.5
**In a steady relationship with a male partner (%)**	49.7***	55.9	48.5	47.2	51.9
HIV status of partner** (%)					
Negative	n.a.*	63.3	66.9	63.7	62.7
Positive		12.5	5.6	11.0	10.9
Unknown		24.2	27.5	25.3	26.4
**Ever tested (%)**	88.9	86.6	91.6	91.0	91.8
**Lifetime median number of tests among ever tested**	3	4	3	3	4
**Returning clients (%)**	n.a.*	23.4***	28.4	35.9	39.6
**Median number of AI partners last 12 months**	3	3	3	3	3
UAI with at least one steady partner**** (%)	63.3	46.1	47.7	55.2	49.3
UAI with at least one casual partner**** (%)	25.3	28.2	26.9	32.4	37.0
**UAI with at least one partner of different/unknown HIV status in the last 12 months (%)**					
Yes	29.7	28.2	32.1	30.5	29.3
No	56.5	69.3	65.5	51.7	61.2
Missing	13.8	2.5	2.4	17.8	9.5
**Main reason for testing**			(n = 289)	(n = 372)	(n = 386)
Sexual risk exposure (%)	-	-	39.8	45.2	39.9
Routine (%)	-	-	30.4	32.0	34.7
Condom stopping in the beginning of a new steady relationship (%)	-	-	12.5	9.4	10.1
STI test (%)	-	-	12.1	5.4	0.0*****
Other (%)	-	-	5.2	8.1	15.3*****

^1^ Males who declared sexual contacts with men, independently of sexual identity or orientation.

* Not available due to incomplete data in the first year of operation. Quality of data in 2005 is somewhat poorer than for the other years because of difficulties around the implementation of the monitoring.

** Among MSM in a steady relationship with a male partner at the time of the consultation.

*** Missing values above 5%.

**** Percentages are calculated among clients who reported anal intercourse with this type of partner in the previous 12 months.

***** A special mobile action on the scene ("Mobile Checkpoint") offering STI rapid testing took place that year and was classified among "other". 6.3% of clients mentioned "Mobile Checkpoint".

The median number of AI partners in the previous 12 months was three, regardless of year.

More than 85% of MSM were repeat HIV testers, and this proportion increased over the years. The lifetime median number of HIV tests among those who were already tested was three or four, depending on the year. As expected, the proportion of returning clients increased (it almost doubled) from 2006 to 2009.

### Sexual risk exposure

Among MSM who reported anal intercourse in the previous 12 months, levels of UAI with steady partners (between 46.1% and 63.3% according to the year) were higher than with casual partners (between 25.3% and 37.0%). Approximately 30% of all MSM had at least one unprotected AI experience with a partner of different/unknown HIV status in the previous 12 months.

### Main reason for testing

The main reason for testing reported by the clients between 2007 and 2009 was “sexual risk exposure” (“risk”, ~40%), second was “routine” testing (“routine”, a little more than 30%), third was “condom stopping in the beginning of a new steady relationship” (“condom stopping”, ~10%), and less than 10% came to the facility for STI testing purposes (Table 3).

In 2009, UAI was the main sexual risk cited (~20% of all MSM), followed by “sperm in mouth” (7.7%).

### Reason for testing and associated characteristics

Only the most relevant findings with significant differences among groups (i.e. “risk”, “routine”, and “condom stopping”) are mentioned below. Table 4 presents the relationship between the main reason for testing and the following variables: median age, currently in a steady relationship with a male partner, median number of AI partners in the previous 12 months, UAI with a partner of different/unknown HIV status in the previous 12 months, past STI, and feelings of depression or sadness during the preceding month.

**Table 4:** **Reasons for testing and associated characteristics among MSM**^1^**clients, 2007-2009**

	**Sexual risk (n = 446)**	**Routine (n = 355)**	**Condom stopping (n = 116)**	**STI (n = 55)**	**Other reasons (n = 105)**	** *p* -values**
New client (%)	70.2	62.0	67.2	60.0	58.1	0.046 (NS)
Median age, years	32	34	29	34	32	0.002*
Median number of past HIV tests	4	4	3	3	3	0.61 (NS)
Currently in a steady relationship with a male partner (%)	41.1	49.3	83.6	38.2	51.4	<0.001*
Median number of AI partners in the last 12 months	4	3	2	3	3	<0.001*
UAI with partner of different/unknown HIV status in the last 12 months (%)	57.3	12.5	23.5	18.0	27.5	<0.001*
Frequent (vs. occasional or never) use of drugs or alcohol before or during sexual intercourse (%)	9.2	5.4	4.3	5.5	7.6	0.178 (NS)
Past STI (%)	41.6	32.2	22.9	65.4	42.6	<0.001*
Felt sad or depressed during last month ^2^ (%)	42.6	32.8	18.5	-	35.7	0.007**

* P-values are flagged as significant relative to the .0055 level computed as Bonferroni correction for overall .05 level with 11 variables.

** Marginally significant after Bonferroni correction.

NS = not significant.

^1^ Males who declared sexual relationships with men, independently of sexual identity or orientation.^2^ Only available since the end of year 2008.

### Sexual risk exposure group

The median age of clients in this group (n = 444) was 32 years, which was younger than “routine” testers and older than “condom stopping” testers. These clients were less frequently currently in a steady relationship (41.1%), than “routine” clients (49.3%) and “condom stopping” clients (83.6%). This group had had the highest median number of AI partners in the previous 12 months (4, compared to 3 for “routine” and 2 for “condom stopping”). Among the clients in this group, 57.3% declared UAI with partners of different/unknown HIV status in the previous 12 months, which was by far the highest rate among the three groups (12.5% of “routine” testers and 23.5% of “condom stopping” clients). Past STI was reported by 41.6% of “risk clients”, compared to 32.2% of “routine” clients and 22.9% of “condom stopping” clients. The clients in this group were also more prone than others to report feelings of sadness/depression in the previous 4 weeks (42.6% compared to 32.8% for “routine” and 18.5% for “condom stopping”). The proportion of feelings of sadness or depression varied marginally between groups (χ² = 12.1, *p* = 0.007).

### Routine testing group

The median age of “routine” testers (n = 355) was 34 years, so they were older than “risk” clients and “condom stopping” clients. Half of the clients in this group were currently in a steady relationship (49.3%), which was far less than the proportion of “condom stopping” clients in a relationship and more than that of “risk” clients. The median number of sexual partners (3) was lower than that of the “risk” group but higher than that of the “condom stopping” group. Only 12.5% of “routine” testers reported UAI, which was the lowest rate among the three groups. The proportion of “routine” testers with a previous STI was 32.2%, which was only more than that of the “condom stopping” clients. One-third (32.8%) of clients in this group felt sad or depressed in the preceding 4 weeks, which was only more than the rate among “condom stopping” clients.

### Condom stopping group

People who came to the facility with the intention to stop using condoms in the beginning of a new steady relationship (n = 116) were the youngest group (29 years). As expected, the proportion of people currently in a steady relationship was high (84%), higher than in all other groups. The median number of AI partners in the previous 12 months was also the lowest (2). UAI was moderate (23.5%), but higher than among “routine” testers. In this group, 22.9% reported having already had an STI, which was the lowest proportion. Only 18.5% of clients in the group reported feelings of depression, which was much lower than in the other groups.

## Discussion

The analysis of the entry questionnaire data from this MSM-friendly Geneva-based VCT facility from 2005 to 2009 has shown an increase in the number of clients and of HIV tests performed, presumably due in part to changes in the organisation of the consultation (acceptance of non-MSM clients, change in business hours, and no need for appointment). The prevalence of new HIV-positive tests among all tests performed was about 2% each year. Clients were a population at particular risk of HIV/STI that had previous experience with HIV testing. The three main reasons for testing were having been exposed to a sexual risk, wanting a routine test, intending to stop using condom in the context of a new steady relationship.

Checkpoint’s clientele increased rapidly since its opening; the number of clients more than doubled between 2005 (n = 245) and 2009 (n = 574). The proportion of new positive HIV tests among all tests performed varied from 1.9% in 2009 to 3.0% in 2007, which is high and similar to the proportion found in the other MSM-specific VCT facilities in Switzerland (3.4% in 2006 in Zurich) [20]. In Amsterdam Checkpoint in 2002–03, the prevalence among MSM was 5.2% and the average prevalence 2.8% [18].

Checkpoint has a good detection rate. In 2009, 28% (9/32) (2005: 10%, 2006: 8.7%, 2007: 11.1%, 2008: 12.1%) of all new HIV cases among MSM living in the canton of Geneva were detected at Checkpoint [26], though a survey conducted among MSM in 2009 in Switzerland (national monitoring) found that most MSM in Geneva had their last test performed by their physician (43.3%) or at other non-MSM-specific VCT facilities of the canton (11.7%) or elsewhere (25.8%). Only 17.5% of MSM reported that they had their last test at Checkpoint [26].

Roughly 90% of MSM tested at Checkpoint had been tested prior to the visit, with a lifetime median number of three prior tests.

The median number of partners in the previous 12 months with whom AI had been practiced (between three and four over the years) and the stable and high proportion (about 30%) of MSM Checkpoint clients reporting UAI with one or more partners of different/unknown HIV status in the previous 12 months suggest that Checkpoint is able to attract clients at high risk of HIV infection. These figures were lower (median of two AI partners and 25% having UAI with partner of different/unknown HIV status) among the Geneva respondents to the national MSM behavioural survey conducted in 2009 [26]. However, we presently underestimate the real proportion of people concerned by this risk exposure (low estimate) because we calculated the proportion without excluding clients who had no anal or no sexual relationship in the previous 12 months and who gave no answer to this question. The capacity to attract high-risk populations was also demonstrated for the similar MSM-specific facility in Zurich [20].

The variety of testing situations encountered in an MSM-specific VCT facility like Checkpoint is illustrated by the distribution of reasons for testing. The three major reasons, accounting for approximately 85% of all reasons, were having been exposed to a sexual risk of HIV transmission, “routine” testing, and wanting to ascertain HIV status in the context of a new sexual relationship. As expected, clients mentioning sexual risk as the main reason for testing were those who had a higher proportion of UAI with a partner of different/unknown HIV status; they were also younger, reported more frequent use of alcohol or drugs before or during sex, and were more likely to report recently feeling sad/depressed. These characteristics have been reported in other studies [27].

Clients who reported “routine testing” as main reason for testing had the lowest proportion of UAI with a partner of different/unknown HIV status, which contrasts with previous results in studies on this topic [28-30]. This may be due to our logic algorithm. However, a non-negligible proportion of ”routine” clients (12.5%) reported UAI with a partner of different/unknown HIV status, and their median number of AI partners was quite high. “Routine” testers are mainly those who come in each year for testing even if they report having taken no sexual risk; they may be in a steady relationship, have not been tested for a while, or want to be reassured (H. Crevoisier, nurse in charge of the facility, personal communication). This population probably deserves special attention and counselling that includes confrontation with their level of risk and assessment of possible psychological suffering.

Clients mentioning the ascertainment of HIV status in the context of a new relationship, including the possibility of stopping condom use, as the main reason for testing also require specific attention and counselling. This group is the youngest and reports the fewest number of partners, but also significant risks and antecedents of STI.

The procedure used at Checkpoint, i.e. answering a computer questionnaire on risks and reasons for testing before the consultation, allows simplification and reinforcement of the individualisation of the counselling, which is especially necessary in the population displaying a wide variety of risk management and risk reduction strategies [31-34]. The “Chat 2009” survey [35], a retrospective qualitative study focusing on the context of new HIV cases in Switzerland, also showed that people are infected in very different ways, which deserves a more specific, personalized approach to risk taking, especially for MSM. Pre-test and post-test counselling may play an important role in helping people better manage the situations in which risks are taken.

The quality of VCT provided at Checkpoint was confirmed in a 2009 national behavioural survey of MSM in Switzerland: among mentioned facilities where MSM go for HIV testing, 79.2% of those having done their last HIV test at Checkpoint Geneva mentioned having received pre-test counselling and 87.5% post- test counselling, the highest rates among all facilities; corresponding figures were 31.5% and 22.2% for test made at a GP's office [4].

Our study has limitations: clients coming back after at least 6 months after a previous visit are considered new clients and complete a new entry questionnaire. Consequently, in the analysis of reasons for testing, as the data from several years are pooled together, the population of reference is the total number of testing situations and not the persons tested since some clients may be included several times.

Another limitation is related to the fact that we conducted secondary analysis of already existing data. The questionnaire used in Checkpoint was designed for counselling purposes and not primarily for research purposes, therefore some questions were not very detailed: for example only one item was used to identify depressive symptoms or psychoactive drugs use; another example is the lack of definition of what constitutes a relationship with a steady partner, left to client's appreciation. However, as literature has shown that the perceived qualification of the relationship is relevant for behavioural adjustment [36,37], we feel that the information collected is valid.

## Conclusions

An MSM-specific VCT facility, such as Checkpoint, is able to respond to various types of testing demand and attracts MSM at high risk of HIV infection, and it should be a useful tool for fighting the HIV epidemic among MSM in Switzerland by attracting the intended population. In fact, many of Checkpoint’s clients reported elevated sexual risk exposure and factors possibly facilitating risk, such as numerous sexual partners, and feelings of depression. The MSM-specific approach offered by the facility is a valuable contribution to the response to the diversity of situations of risk exposure among MSM.

## Competing interests

The authors declare that they have no competing interests.

## Authors’ contribution

CG: data analysis, writing of the article. AJ: data analysis supervision, participation in the writing. HB: data analysis, draft article reading and final approval. TH: conceptualization of the study, draft article reading and final approval. VJ: participation in the data collection organization. MH: conceptualization of data collection, draft article reading and final approval. HC: data collection and data processing, draft article reading and final approval. PS: draft article reading and final approval. FDA: conceptualization and direction of the study, participation in the writing. All authors read and approved the final manuscript.

## Pre-publication history

The pre-publication history for this paper can be accessed here:

http://www.biomedcentral.com/1471-2458/12/422/prepub
